# Balancing benefits and risks of exercise in pregnancy: a qualitative analysis of social media discussion

**DOI:** 10.1136/bmjsem-2024-002176

**Published:** 2024-10-11

**Authors:** Emilie J M Côté, Madeleine Benton, Rachael Gardner, Rachel Tribe

**Affiliations:** 1 Department of Women and Children’s Health, School of Life Course & Population Sciences, King's College London, London, UK; 2 Department of Psychological Medicine, Institute of Psychiatry, Psychology and Neuroscience, King's College London, London, UK; 3 Reproductive Health and Childbirth, Guy's and St Thomas' NHS Foundation Trust, London, UK

**Keywords:** Exercise, Pregnancy, Behaviour

## Abstract

Exercise improves pregnancy outcomes, but few pregnant individuals meet physical activity guidelines. The main objective of this study was to explore the perception of exercise during pregnancy using posts and comments on Reddit, a large social media platform. Relevant user-generated posts and comments were identified on Reddit by searching systematically for a combination of keywords related to pregnancy and exercise. A dataset of 120 posts and 2892 comments was randomly selected for reflexive thematic analysis. Three themes relating to perceptions of benefits and risks to exercise in pregnancy and how they influence decision-making were generated: (1) perceived benefits of exercise: ‘We all know that exercise is good for us’; (2) perceived risks of exercise: Exercise as ‘off limits’; and (3) information seeking and decision-making: ‘I’m kinda stumped on exercise’. While the benefits of exercise during pregnancy are well recognised among Reddit users, perceived risks significantly influence their decisions to start, continue, adjust or stop exercising. Healthcare providers play a crucial role in guiding these exercise choices, emphasising the need for them to provide evidence-based advice and support pregnant individuals in achieving optimal physical activity levels. Addressing misinformation and providing supportive counselling can help pregnant individuals navigate the complexities of exercise during this critical period.

**WHAT IS ALREADY KNOWN ON THIS TOPIC**

- Exercise in pregnancy is beneficial. Current guidelines recommend that pregnant individuals do at least 150 min of moderate-intensity cardiovascular exercise and two full-body strength training sessions every week. Still, most women reduce, stop or do not start exercising due to safety concerns which may stem from contradictory information found on social media or from medical or fitness professionals. Insufficient scientific evidence exists to address many of these concerns.

**WHAT THIS STUDY ADDS**

- We used social media data to identify perceived benefits and risks of exercise in pregnancy and to explore women’s experiences of sourcing information and making decisions about exercise.

**HOW THIS STUDY MIGHT AFFECT RESEARCH, PRACTICE OR POLICY**

- We identify commonly held safety concerns and summarise the available evidence. Clinicians are trusted and should provide patients with practical and nuanced evidence-based advice about exercise during pregnancy.

## Introduction

 Exercise during pregnancy has benefits that include improved mental health, reduced gestational weight gain and reduced risk of pregnancy complications.^1^ Over 30 ‘exercise in pregnancy’ guidelines have been published, most evidence-based and of acceptable methodological quality.^2 3^ Clinical and public health guidance has evolved from recommendations to limit activity to recent guidelines encouraging pregnant individuals to exercise.^4^ For example, the UK chief medical officer recommends at least 150 min of moderate-intensity activity and two full-body strength training sessions per week.^5^ For those with pregnancy complications such as placenta praevia or ruptured membranes, exercise restriction is still viewed as the safer option in the absence of evidence to the contrary.^6^

Most individuals reduce physical activity while pregnant, with high rates of sedentarism.^7^ Systematic reviews have identified barriers to exercise, including pregnancy-related symptoms, time constraints, perception of already being active, lack of motivation, mother-child safety concerns and lack of advice and information.^8^,^10^

Women increasingly use social media to gather information on exercise during pregnancy and seek peer support.^11 12^ Qualitative researchers can use data generated by groups who may not otherwise engage with traditional research methods, thus improving ecological validity.^13^ Previous studies on social media content about exercise in pregnancy involve small participant numbers and have identified mixed messages about benefits and safety.^14^,^16^ This study aimed to explore contributors’ understanding of exercise in pregnancy on a large social media platform, Reddit.

Reddit is a large discussion-based social media platform on which users engage in open, publicly available text and image-based discussions. The platform’s structure allows for in-depth exchanges in themed communities, or ‘subreddits’, where users post, comment and respond to others’ comments. This differentiates Reddit from other social media platforms where interactions are limited to personal networks (eg, Facebook), have specific formats (eg, short text on Twitter/X or images with captions on Instagram) or have limited subject matter (forums like Mumsnet). These factors make Reddit an ideal platform for exploring nuanced user-generated content on the topic of exercise during pregnancy.

## Methods

### Research design

A qualitative research design using data collected from social media posts and a reflexive thematic approach was used.

### Data collection

Reddit was searched using a script through its application programming interface (API) on 1 February 2024 with keyword combinations relating to pregnancy and exercise (online supplemental appendices A and B). Results were sorted by ‘Relevance’ and ‘All Time’. The top 50 posts were included for each search, and duplicates were removed. Results were screened and included if the post referred to exercise in pregnancy, excluding posts referring only to pelvic floor health. Due to the search strategy, only English language results were retrieved. A subset of 120 posts and their comments were selected randomly using Excel’s random number generator for analysis.

### Reflexivity

We continuously reflected on how our personal values, beliefs and assumptions influenced the research process. The team had varying professional backgrounds, clinical training and research experience, and some of the team had experienced multiple pregnancies. They had a range of engagement with physical activity, from walking to regular vigorous exercise. The team included an obstetrician (EJMC), chartered psychologist (MB), midwife (RG) and scientist (RT). Authors EJMC and MB have experience using thematic analysis and trained in NVivo, and MB has extensive experience in qualitative research methods in the preconception, pregnancy and postnatal periods.

### Data analysis

Reflexive thematic analysis was used for its flexible approach to data analysis. It is situated within an interpretive paradigm, where data interpretation is subjective rather than objective, knowledge is socially constructed and researchers are active participants in knowledge generation.^17^ Six steps outlined by Braun and Clarke were followed: familiarisation with data, coding, generating initial themes, reviewing themes, naming themes and writing the report.^18^ EJMC coded the data in NVivo, developed the themes and discussed and refined them reflexively with MB. Data excerpts were selected to illustrate the themes and subthemes.^13 18^

### Equity, diversity and inclusion

As of March 2024, Reddit was the eighth most visited website globally, with the USA accounting for 60% of visits.^19^ Gendered language is used by Reddit contributors in the dataset. The experience of male or non-binary gestational parents is not discussed as it was not represented in this dataset.

### Patient and public involvement

The public was not involved in the planning of this study. Pregnant and postpartum individuals, along with fitness and healthcare professionals, were consulted after the initial generation of themes. Six participants in an antenatal aqua aerobics fitness class responded to an online freeform questionnaire asking about their personal experiences and opinions of exercise during pregnancy. One aqua aerobics instructor, two CrossFit instructors, two obstetricians, two midwives and two student midwives gave feedback after a presentation of the study’s preliminary findings. This consultation contributed to the discussion of clinical implications.

### Ethical and data considerations

Reddit granted permission to use its API to collect data (January 2024). Contributor usernames were removed to preserve privacy.

Research using data from social media mining sits in a grey area which requires ethical considerations beyond approval by a research ethics committee.^20^ We considered that data collection was ethically acceptable in this case as several factors suggested that users understood that the content they posted was public. All users must agree to Reddit’s Privacy Policy when registering for an account. Additionally, Reddit allows for moderators to make subreddits public or private, with the private option chosen when users do not wish for discussion to be visible on the internet outside of users of that subreddit. The content used in this study came exclusively from public subreddits, suggesting that users do not consider it to be sensitive content. Given these considerations, we took the view that seeking informed consent from individual users was impractical and disproportionate to the minimal risk of harm.

## Results

### Data description

The search generated 4877 unique links to Reddit posts, of which 1121 (23%) were relevant to the research question. These were posted by 1011 unique Reddit contributors from 2010 to 2024 and include 26 323 comments.

Posts were concentrated on fitness or pregnancy-related subreddits (online supplemental appendix C). The 120 posts in the final dataset were authored by 120 contributors and contained 2892 comments. The date range and subreddits covered were similar to the wider dataset.

### Thematic analysis

**Figure 1:**
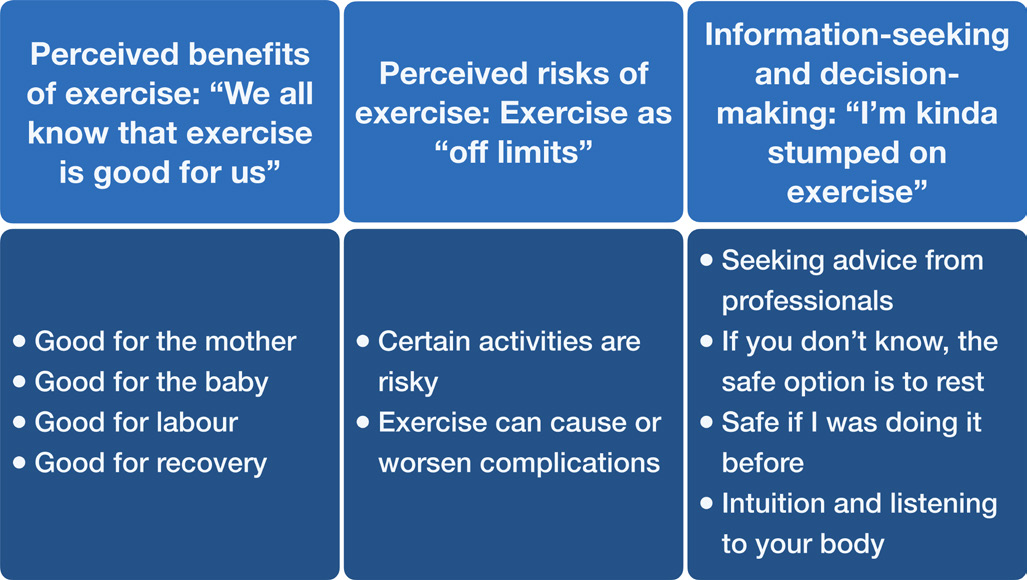
Themes relating to exercise in pregnancy—benefits, risks and decision-making. Thematic analysis of 120 posts and 2892 comments from the Reddit social media platform.

The analysis generated three themes (figure 1).

#### Theme 1. Perceived benefits of exercise: ‘*We all know that exercise is good for us*’

##### Good for the mother

The benefits of exercise were widely discussed. Contributors stated that exercising during pregnancy made them feel good ‘*mentally and physically*’. By being ‘*in good shape*’, knock-on benefits to exercising were highlighted, including ‘*higher energy levels*’, leading them to ‘*maintain […] activity levels during the pregnancy*’ to improve sleep and preserve muscular strength and cardiorespiratory fitness. Exercise helped manage pregnancy symptoms, including nausea, back pain, pelvic pain, hip pain, sciatica and other ‘*aches and pains*’.

Many also discussed mental health benefits. They highlighted that exercise helped improve well-being and ‘*balance out hormonal swings*’. One contributor explained:

I cried a lot this pregnancy, will do anything and everything to keep PPD [post-partum depression] at bay. And that’s almost the main reason I am pushing myself to be more regular now.

While some contributors discussed the fact that physical activity reduces the risk of pregnancy complications such as gestational diabetes, they did not mention that this motivated them to exercise.

##### Good for the baby

Exercise was commonly perceived as ‘*beneficial for the baby*’. Contributors cited the growth of a healthy placenta and baby or improved circulation to the developing brain. The notion of contributing to their baby’s well-being was motivating for some:

I am a gym rat big time, but my number one motivator is for my LO [little one] to be healthy.

However, failing to meet self-imposed exercise goals resulted in contributors describing negative psychological impacts:

I just wanna cry—I feel like I’m ruining my health and baby’s health.

##### Good for labour

Many women stated that exercise could help them ‘*prep[are] for labour*’, starting with getting ‘*the baby in a good position*’. A minority of women believed that walking could help induce labour. Women who stated they felt physically fit reported an improved experience in relation to labour duration, pushing efficiency and stamina:

I was able to push actively for almost three hours, that I had the stamina and physical capacity for that. If I hadn’t have been so ﬁt, I think I would have ended up getting a csection.

Women explicitly compared the psychological skills developed through exercise to those pertinent to labour:

Working out is a great way to become in tune with your body. All the things that get you through a heavy lift can be applied to childbirth. Mind and body connection, knowing which muscles to engage, and general trust in your body.

##### Good for recovery

Women discussed using exercise for postnatal ‘*recovery’*, with some emphasising a swift return to physical activity. Physical appearance was often mentioned as an important marker of postnatal recovery. Women described apprehension regarding weight gain during pregnancy, which paralleled a desire to lose weight rapidly post partum. Attaining a pre-pregnancy physique was widely regarded as desirable, although not universally achievable:

I worked with one of the ﬁttest women I have ever met. […] She continued exercising right up until she gave birth. She had an easy birth from what I understand, and four days later she was dressed in her pre-pregnancy clothes and headed out to the club. I was amazed.

Prevention of diastasis recti and safeguarding of pelvic floor health were frequently discussed. Some women exercised to mitigate the risk of developing these issues, but more expressed worry that exercise might exacerbate them.

### Theme 2. Perceived risks of exercise: Exercise as *‘off limits*’

#### Certain activities are risky

Contributors frequently mentioned perceived risks of exercise in pregnancy, which we have categorised into six domains—high intensity, high impact, trunk and core, trauma, environment, and movements and positions—and summarised with illustrative quotes in online supplemental appendix D. Contributors sometimes identified explanations for, and consequences to, the ‘*risky*’ activities. Still, in most instances, these remained unspecified.

Advice regarding risk avoidance was often contradictory: squats were recommended by many, while others advised against them and recommended lunges. One contributor warned against unilateral movements in general and lunges in particular. This pattern repeated itself for core work. Contributors reported that abdominal exercises should be approached carefully during pregnancy, often due to a fear of exacerbating the development of diastasis recti abdominis (stretching of the linea alba leading to an increased inter-rectus distance). However, advice was conflicting:

I think your safest bet is to avoid abdominal exercises that work the rectus abdominis. Focus more on strengthening the transverse abdominis.

You should just avoid exercises that target the TVA [transversus abdominis].

Women frequently sought advice from each other through their posts and comments—they mostly accepted recommendations without in-depth questioning or discussion. There was an overall sense of trust in the lived experiences of peers.

#### Exercise can cause or worsen complications

Discussion surrounding exercise risks during pregnancy also included pregnancy complications in two ways. First, contributors considered how exercise might directly cause a complication in a healthy pregnancy, for example, a spinal twist in yoga causing placental abruption or exposure to heat causing spina bifida in the fetus.

Previous experience of complications, including pregnancy loss, also seemed to influence decision-making. Women at risk of miscarriage were commonly told or independently decided to stop exercising: one participant with previous losses reported that she ‘*stopped working out [after] the ﬁrst two losses out of fear and sheer exhaustion*’.

Second, women discussed that having a pregnancy complication could mandate a reduction or cessation of physical activity. In some cases, the complication was seen as an absolute contraindication to exercise:

My dr told me I could keep lifting heavy and doing what I was already doing, but to maybe go more like 90% than 100%. […] Then I got placenta previa and had to stop all high impact activities and lifting everything over 15 lbs.

Once they had experienced a complication, women discussed exercise as something that they should be ‘*cleared*’ for by a medical professional.

### Theme 3. Information seeking and decision-making: ‘I’m kinda stumped on exercise’

#### Seeking advice from professionals

Women regarded healthcare professionals as trustworthy sources of information regarding exercise during pregnancy. While contributors shared advice on Reddit, they also emphasised the importance of deferring to medical guidance. Medical advice was often prioritised over recommendations from fitness professionals like coaches, as doctors and midwives were perceived to offer personalised advice:

I think your doctor will know best. Everyone is different so they’ll be able to give the best advice speciﬁc to you.

Women derived a sense of safety from following medical advice, even when they recognised that it might be excessively cautious:

[My doctor] was very anti anything that involved bouncing/feet leaving the ground, so I stopped running shortly into my pregnancy. I had a lot of people tell me my doctor was wrong but mentally, it felt like the right choice to just listen to them.

Some individuals chose their healthcare professionals with exercise in mind: one woman whose ‘*local midwife practice still has a 25lb lifting limit in their pregnancy guideline handout*’ described booking her pregnancy with a ‘*pro-exercise*’ doctor instead.

#### If you do not know, the safe option is to rest

In the absence of personalised advice, rest was often seen as the safest option. For example, one contributor limited activity after the diagnosis of a growth-restricted baby:

The doc told me I could keep working out but psychologically I just couldn’t. […] I just don’t think I can do my workouts without feeling guilty or anxious.

Women reported feeling ‘*nervous*’ during high-intensity activity or waiting to exercise ‘*until at least after my anatomy scan just because I’m paranoid*’. Women also advised others to ‘*play it safe and stick to low-impact and low-intensity exercise*’.

However, not everyone agreed that rest is the safest option:

These recommendations act like there is NO amount of acceptable risk tolerance. Like, if there’s even a minuscule chance that running could cause you to fall, you shouldn’t do that. This totally overlooks the beneﬁts: for example, my mental health suffers tremendously when I don’t exercise. I’m more than willing to trade a small risk of falling for improved mental health—which is also a safety issue for myself and my baby.

Surprisingly, this was the only comment to explicitly frame decision-making about exercise in pregnancy as a risk-benefit equation.

#### Safe if I was doing it before

Many women who exercised regularly preconception expressed a desire to continue during pregnancy. They appeared to have fewer concerns about exercise safety and believed that ‘*you can keep doing what you’re doing minus the obvious exceptions*’. Users also reported that their doctors supported the continuation of established pre-pregnancy workout routines.

Contributors also commonly expressed that a pregnant person should not start a new exercise regimen. One recounted the same advice from a doctor and was told not to increase weights for ongoing exercise. However, lower intensity exercise such as walking and yoga was generally perceived as safe to start during pregnancy, with one woman explaining that it would not ‘*overwhelm your body*’.

Some contributors provided justifications for the caution surrounding new physical activity. They explained that individuals unfamiliar with an exercise modality might misinterpret ‘*body signals*’, leading to inadequate adjustments and possibly endangering the pregnancy.

#### Intuition and listening to your body

Understanding body signals to exercise safely was a common theme. Most participants trusted that they could use this strategy effectively:

Listen to your body and remember that your body is still yours, and you know it best!

Contributors occasionally specified which body signals should be considered, citing ‘*DR [diastasis recti], pain, dizziness, bleeding after lifting, and braxton hicks*’. One self-identified fitness professional commented that individuals should assess whether the workout made them ‘*feel better or depleted*’.

Some women who identified as active reported that they struggled to adjust during pregnancy. One woman described pushing through pain to complete her workout. Commenters chided her, with one explaining that it could be ‘*very dangerous*’ to ‘*fixat[e] on the ‘plan’ instead of listening to your body*’.

The advice to ‘*listen to your body*’ also came from medical professionals. This was described as ‘*really not comprehensive enough for pregnancy*’ by one woman who felt that ‘*just because you can still do it, doesn’t mean you should*’. Another contributor was pleased with her high-risk obstetrician’s advice to ‘*do whatever exercises I wanted as long as they felt comfortable to my body*’. She passed on the advice to another contributor, clarifying that ‘*if you’re really in tune with your body, go for it!*’.

## Discussion

Three themes were generated from the analysis, centring on the benefits of exercise in pregnancy, its perceived risks and how contributors gather information to inform decision-making. Engaging with this information and understanding contributors’ concerns can support clinicians’ approach to counselling pregnant individuals about exercise. Reddit contributors recognised the benefits of exercise for the pregnant person, the baby, labour and recovery. These benefits are generally well supported by existing evidence.^1^ Awareness of these drivers could inform the focus of public health messaging about exercise during pregnancy.

Contributors labelled certain activities as risky during pregnancy. We summarise recent evidence for each identified risk in table 1, but research is lacking in key areas. For example, there is scarce evidence on the safety of high-intensity exercise (>90% maximum heart rate) during pregnancy or vigorous-intensity exercise during the first and second trimesters. Similarly, many of the perceived risks and recommendations that women share about the impact of exercise on diastasis recti appear unfounded. Overall, the studies investigating the safety of specific pregnancy exercises conclude that they have a neutral to beneficial impact on health outcomes.

**Table 1:** Evidence for exercise perceived as ‘risky’ by Reddit users

Activity	Evidence
**Intensity**	
High intensity	Moderate to vigorous activity after 12 weeks does not increase the risk of congenital abnormalities.^25^Vigorous exercise in the third trimester reduces the risk of prematurity.^26^ Continued heavy resistance training is associated with reduced pregnancy and postpartum complications.^27^
Straining	There is no evidence of harm from the Valsalva manoeuvre during resistance training on blood pressure, placental blood flow or postpartum pelvic floor disorders.^27 28^
**Impact**	
High impact (bouncing, jumping, running)	High-impact aerobics combined with pelvic floor muscle training improves pelvic floor muscle tone and reduces the impact of postpartum urinary incontinence.^29 30^ High-impact exercise in pregnancy is associated with a lower caesarean birth rate.^31^
**Trunk and core**	
Abdominal exercises and stretching	Prenatal core exercises reduce pain symptoms, improve the chance of vaginal birth and reduce the risk of perineal trauma.^32 33^ Abdominal exercise in pregnancy has a neutral to positive impact on DRA antenatally and postnatally. Some exercise programmes with favourable outcomes included crunch-based exercises. TVA activation exercises were also included but were not found to be helpful in isolation.^34^,^36^
**Trauma**	
Activities with a risk of falls (eg, bouldering, skiing, box jumps)	The rate of exercise-related injury in pregnancy is 4.1:1000 exercise hours. 55% of injuries were bruises and scrapes.^37^ Major falls leading to hospitalisation are linked to increased risk of lower limb injury, preterm birth, placental abruption, induction of labour, caesarean section, fetal distress and fetal hypoxia.^38^
Abdominal pressure/trauma (eg, from barbell)	There is no difference in injury rates between those who continue Olympic weightlifting during pregnancy and those who do not.^27^Babies born to individuals who engage in regular resistance training have higher birth weights.^39^
**Environment**	
Heat (eg, from exertion, hot yoga, hot weather, heated pool)	Environmental heat exposure is associated with adverse maternal and neonatal outcomes.^40^ Pregnant individuals maintain a safe core body temperature during^41^,^43^: Aerobic exercise at 90% maximum HR for ≤35 min at ≥25°C air temperature and 45% RH. Aerobic exercise at moderate intensity for ≤45 min at 32°C and 45% RH. Exercise in 33.4°C water for ≤45 min. Rest in a dry sauna at 70°C at 15% RH for ≤20 min. 40°C bath for ≤20 min. Hot yoga session for 60 min at 35.4°C and 56.7% RH.
High altitude	Limited heterogeneous literature does not identify significant risks with exercising at altitude in healthy pregnancies.^44 45^
**Movements and positions**	
Lying supine	Maternal vital signs and fetal heart rate remain normal through a yoga class that includes supine postures.^46^ Acute bouts of supine exercise are associated with transient fetal HR abnormalities and reduced umbilical blood flow. This is not associated with reduced birth weight.^47^
Lying prone	There is no change in haemodynamic parameters when resting in a prone position using a pillow.^48^ No exercise-specific evidence was found.
Inversions	No evidence was found.
Squats	Habitual squatting during ADLs is associated with a shorter second stage of labour and a higher rate of spontaneous vaginal birth.^49 50^
Unilateral movements	No evidence was found.
Backbends	Maternal vital signs and fetal heart rate remain normal through a yoga class, including a backbend.^46^
Hanging	No evidence was found.
Twisting	No evidence was found.

ADLsactivities of daily livingDRAdiastasis recti abdominisHRheart rateRHrelative humidityTVAtransversus abdominis

Contributors explained their approach to finding information and making decisions about exercise during pregnancy, citing doctors and midwives as trusted sources of individualised advice. They reported advice from healthcare providers and each other to listen to their bodies, which is an interoceptive process that may be improved in those with higher pre-existing fitness levels.^21^ This may be an additional barrier to meeting exercise guidelines in sedentary pregnant individuals, as they self-select activities with lower energy expenditure.^22^ In addition, contributors reported limiting activity when there was doubt about safety, often in response to medical advice. Some guidelines provide lists of absolute contraindications to exercise, which are consensus rather than evidence based (eg, asymptomatic placenta praevia after 28 weeks’ gestation).^2 6^ This cautious approach may contribute to high rates of sedentarism during pregnancy.

### Clinical implications

Increasing numbers of individuals are turning to social media for pregnancy information, advice and support.^12^ We have identified common concerns about exercise safety which are not supported by evidence. Doctors and midwives should remain up to date with current guidance and discuss misinformation with their pregnant patients, supporting them to meet physical activity recommendations and improve lifelong health.

### Strengths and limitations

Given its structure and research-friendly terms of use, Reddit is a useful resource for spontaneously generated social media data. Our search strategy used a range of keywords to maximise the breadth of the thematic analysis. The dataset is limited by its bias towards people who engage in social media discussion of exercise during pregnancy: the most represented subreddit is ‘fitpregnancy’, which is described as a space for ‘motivating and inspiring pregnant people to stay healthy and fit throughout’.^23^ Many of the comments included in this study were thus from users who were seeking out advice and support on how to remain active throughout their pregnancy. Other subreddits contained commentary from self-reportedly inactive users, who may have different beliefs about benefits and risks of exercise which were under-represented as they focused their discussion on barriers to exercise. Reddit users are also predominantly from English-speaking countries and have access to the internet, with fewer users from low- to middle-income countries. This may have influenced the data we collected, as culture impacts how exercise in pregnancy is perceived. For example, a comparison of Australian and Chinese pregnant women found differences in attitudes, perceived norms, intentions to exercise, physical activity levels, choice of activity and barriers to exercise in pregnancy.^24^ Interventions to improve uptake of exercise in pregnancy would therefore benefit from research into culturally informed beliefs which may need to be addressed in the population of interest. Despite these limitations, the dataset provided rich insights into perceptions of exercise in pregnancy.

## Conclusion

This study demonstrates the impact of perceived risks and benefits on pregnant individuals’ decisions regarding physical activity. Many of these perceived risks lack empirical evidence and are perpetuated through historical expert opinion, often in contemporary medical guidelines and social media discourse. Given the growing recognition of sedentary behaviour as a primary risk factor for chronic disease, it is imperative that we rigorously investigate exercise during pregnancy so that pregnant women and people can make informed decisions. There is an opportunity to leverage social media for public health messaging and better reach this population.

## supplementary material

10.1136/bmjsem-2024-002176 online supplemental file 1

## Data Availability

No data are available.
