# Supplementary material for: Balancing benefits and risks of exercise in pregnancy: a qualitative analysis of social media discussion
Source: BMJ Open Sport Exerc Med. 2024 Oct 11;10(4):e002176. doi: 10.1136/bmjsem-2024-002176 (PMC11481124; doi:10.1136/bmjsem-2024-002176)
Supplement: online supplemental file 1 [file bmjsem-10-4-s001.pdf]

Appendix A

Pregnancy search terms

Pregnancy, antenatal, prenatal, first trimester, second trimester, third trimester, birth, labor, labour, delivery

Exercise search terms

Physical activity, fitness, aerobic, anaerobic, cardio, strength, yoga, pilates, barre, crossfit, hiit, interval training, running, cycling, jogging, walking, dance, martial arts, training, sport, swimming, flexibility, endurance, rehab, hiking, weights

Appendix B

```
import pandas as pd
import praw
import prawcore
import time
from itertools import product

# Reddit credentials
Redacted

# Initialize PRAW
reddit = praw.Reddit(client_id=client_id,
client_secret=client_secret,
user_agent=user_agent,
username=username,
password=password)

def search_reddit(keyword1, keyword2, sort='relevance', time_filter='all'):
urls = []
search_query = f"{keyword1} {keyword2}"
for submission in reddit.subreddit("all").search(search_query, sort=sort,
time_filter=time_filter, limit=50):
if 'reddit.com' in submission.url:
urls.append((submission.url, keyword1, keyword2))
return urls

def process_csv(file_path, output_file):
all_urls = []
# Read the CSV file
df = pd.read_csv(file_path)

# Create all combinations of keywords from column_1 and column_2
```

```
combinations = product(df['column_1'], df['column_2'])

# Iterate through each combination and perform a search
for keyword1, keyword2 in combinations:
# Skip the search if either keyword is empty or None
if pd.isnull(keyword1) or pd.isnull(keyword2) or not keyword1.strip() or not
keyword2.strip():
continue

try:
all_urls.extend(search_reddit(keyword1, keyword2, sort='relevance',
time_filter='all'))
time.sleep(3) # Sleep for 1 second between requests
print(f"{keyword1} {keyword2}")
except prawcore.exceptions.TooManyRequests as e:
print("Rate limit exceeded, sleeping for 60 seconds.")
time.sleep(60) # Sleep for 60 seconds if rate limit is hit

# Process for unique URLs and associated search terms
unique_results = {}
for url, keyword1, keyword2 in all_urls:
if url not in unique_results:
unique_results[url] = (keyword1, keyword2)

# Prepare data for CSV
final_data = [{'URL': url, 'Keyword1': terms[0], 'Keyword2': terms[1]} for url, terms
in unique_results.items()]

# Save to CSV file
pd.DataFrame(final_data).to_csv(output_file, index=False)

print(f"Unique URLs with search terms saved to {output_file}")

process_csv('reddit_search_keywords_limited.csv', 'unique_urls.csv')
```

Appendix C – Common subreddits

"fitpregnancy" focuses on fitness in the perinatal period.

"xxfitness" focuses on fitness for women.

"BabyBumps", "pregnant", and "PregnancyUK" focus on pregnancy.

"beyondthebump" is for discussion of the postnatal period.

"yoga", "Purebarre", "pilates", and "crossfit" focus on those specific exercise modalities.

Appendix D

Table 2: Summary of activities considered to be "risky" in pregnancy based on analysis of 120 posts and 2,892 comments from the Reddit social media platform

Activity	Quotes	Summary of Reddit content	
		Perceived risks	Recommendations
**INTENSITY**			
High intensity	"Ob gyn tells me to avoid jumping and HIIT; my CF coach says that this shouldn't be an issue, that I can continue BAU; and obvs the internet has an opinion on everything…"	Not mentioned, harm to mother, reduced oxygenation to baby, risk of injury	Avoid, do same activity at lower intensity, switch to another activity type, do prenatal-specific exercise, limit heart rate, remain able to speak in full sentences during activity
Straining	"Remember you cant brace like before."	Not mentioned, transient increase in blood pressure, harm to fetus	Avoid
**IMPACT**			
High impact (bouncing, jumping, running)	"I feel awful about the baby bouncing around, makes me think I'm going to cause the umbilical cord to wrap around his neck or something"	Not mentioned, breast/hip/back/round ligament/joint pain, pelvic floor pressure, fall risk, depletion of iron stores, overheating, hypoglycaemia, tangled umbilical cord, harm to placenta, worsen subchorionic	Switch to low impact movement (e.g. swimming, walking, pregnancy-specific), use belly band, hydrate, eat well, reduce intensity, stay close to home, have someone with you

		haematoma, miscarriage, harms baby's development	
**TRUNK AND CORE**			
Abdominal exercises and stretching	"No core work from very early on. Slowly reducing weights so I don't feel my core engaging"	Not mentioned, antenatal or postnatal diastasis recti abdominis (DRA), discomfort, belly in the way, uterine cramping	Avoid, consult professional for appropriate exercises, do prenatal-specific exercise, avoid targeting the rectus abdominis (RA), strengthen the tranversus abdominis (TVA), avoid targeting the TVA, avoid coning, avoid front-loading, avoid flexion, avoid movements that cause a pressure sensation
**TRAUMA**			
Activities with risk of falls (e.g. bouldering, skiing, box jumps)	"I'm usually pretty sure-footed but when I was starting to get big with my first it was like every day I was more off kilter and I constantly felt like I was on the verge of falling down. And falling during pregnancy can be DISASTROUS."	Not mentioned, harming the baby, harming the placenta	Avoid, fine if it happens during the first trimester, substitute with other activity, use safety gear, continue but stick to your skill level

Abdominal pressure/trauma	"minimize movements with a barbell that could hit the belly, so no barbell snatch nor clean"	Non-specific harm to mother and baby	Avoid, use full-body harness for climbing, do martial arts with trusted partner, use dumbells instead of a barbell, lower the weight to control the bar path
**ENVIRONMENT**			
Heat (from exertion, hot yoga, hot weather, heated pool)	"I'm an advanced practitioner of yoga and love hot yoga — I didn't even consider returning to my normal practice"	Not mentioned, maternal tachycardia, spina bifida, dizziness and syncope	Avoid, hydrate, cool down, stay close to home, reduce intensity, fine to do
High altitude	"Avoid trekking or exercising 2,500m above sea stage – this is due to the fact you and your toddler are at threat of altitude sickness."	Altitude sickness	Avoid
**MOVEMENTS AND POSITIONS**			

Lying supine	"Your vena cava gets compressed in second trimester so no lying on your back/minimize"	Not mentioned, diastasis recti, cutting the circulation to the baby (mentioned as a myth), heartburn, compression of the vena cava and aorta, hypotension, reduced blood supply to the placenta, reduced fetal oxygenation	Avoid, do same movement at an incline
Lying prone	"You will eventually need to modify anything that stretches your abs too much, some twists, and lying on your belly or back."	Not mentioned	Avoid, substitute another movement
Inversions	"I will say even now I can still do handstands comfortably- it just makes everyone else uncomfortable - so I stopped.	Not mentioned, risk of falling, heart strain, heartburn, dizziness	Avoid
Squats	"Thing is now, she is concern on what exactly she will be doing during the 2nd and 3rd trimester. We know she wont be doing any box jump, skipping or squat under parallel position"	Not mentioned, coning, pelvic pressure	Avoid, reduce weight, don't squat under parallel, switch from front squat to other squats, switch to unilateral movements
Unilateral movements	"Also be careful of one sided loaded moves as you progress - such as lunges and running."	Not mentioned	Avoid
Backbends	"Camel is a pretty intense backbend and	Not mentioned	Avoid

	not recommended for most pregnant women"		
Hanging	"Im 22 weeks now and can no longer do […] bar hangs"	Not mentioned, stretching the abdomen, rib strain, excessive core activation	Avoid, substitute another movement, do same movement at a lower intensity
Twisting	"Be careful. Someone I know had a placenta tear during her last trimester doing a supine twist."	Not mentioned, placental abruption	Avoid, limit range of motion, do 'open' but not 'closed twists'
